# Impact of homologous and non-homologous recombination in the genomic evolution of Escherichia coli

**DOI:** 10.1186/1471-2164-13-256

**Published:** 2012-06-19

**Authors:** Xavier Didelot, Guillaume Méric, Daniel Falush, Aaron E Darling

**Affiliations:** 1 Department of Infectious Disease Epidemiology, Imperial College, Norfolk Place, London W2 1PG, UK; 2 College of Medicine, Swansea University, Swansea, SA2 8PP, UK; 3 Max Planck Institute for Evolutionary Anthropology, Deutscher Platz 6, Leipzig 04103, Germany; 4 Genome Center, University of California, Davis, CA 95616, USA

## Abstract

**Background:**

*Escherichia coli* is an important species of bacteria that can live as a harmless inhabitant of the guts of many animals, as a pathogen causing life-threatening conditions or freely in the non-host environment. This diversity of lifestyles has made it a particular focus of interest for studies of genetic variation, mainly with the aim to understand how a commensal can become a deadly pathogen. Many whole genomes of *E. coli* have been fully sequenced in the past few years, which offer helpful data to help understand how this important species evolved.

**Results:**

We compared 27 whole genomes encompassing four phylogroups of *Escherichia coli* (A, B1, B2 and E). From the core-genome we established the clonal relationships between the isolates as well as the role played by homologous recombination during their evolution from a common ancestor. We found strong evidence for sexual isolation between three lineages (A+B1, B2, E), which could be explained by the ecological structuring of *E. coli* and may represent on-going speciation. We identified three hotspots of homologous recombination, one of which had not been previously described and contains the *aroC* gene, involved in the essential shikimate metabolic pathway. We also described the role played by non-homologous recombination in the pan-genome, and showed that this process was highly heterogeneous. Our analyses revealed in particular that the genomes of three enterohaemorrhagic (EHEC) strains within phylogroup B1 have converged from originally separate backgrounds as a result of both homologous and non-homologous recombination.

**Conclusions:**

Recombination is an important force shaping the genomic evolution and diversification of *E. coli*, both by replacing fragments of genes with an homologous sequence and also by introducing new genes. In this study, several non-random patterns of these events were identified which correlated with important changes in the lifestyle of the bacteria, and therefore provide additional evidence to explain the relationship between genomic variation and ecological adaptation.

## Background

Recombination is a fundamental process of bacterial evolution, capable of influencing the integrity of species [1-3]. Two types of recombination are typically distinguished: homologous recombination, where a fragment of a genome is replaced by the corresponding sequence from another genome [4], and non-homologous recombination, which causes genetic additions of new material and is also called lateral gene transfer (LGT) [5]. These two types of recombination may in fact often happen simultaneously, but they are usually studied separately because of the very different signatures they produce on the genomic sequences. Both homologous and non-homologous types of recombination are key elements of the evolution of bacteria and can be linked to variations in fitness, and thus ecologies and lifestyles. There is indeed an ecological component in bacterial recombination, in the sense that bacteria with overlapping living environments, reservoirs or hosts (i.e., “overlapping ecologies”) will have more opportunities for genetic exchange than species or lineages living in drastically distinct environments. Recombination is therefore clearly conditioned by ecology, but conversely it is probable that recombination often drives ecological changes, for example by allowing favourable innovations to be exchanged by separate lineages adapting to a same lifestyle [3,6].

*Escherichia coli* is a good example of an environmentally versatile and adaptable bacterial species. It encompasses some strains able to live commensally with their host and others causing a relatively wide variety of disease symptoms, from diarrhoea or renal failure to meningitis [7]. On top of this commensal versus pathogen duality, which may not represent a strict categorization, *E. coli* can be found in a wide range of hosts, as well as secondary non-host environments such as water, soils or plants [8,9], in which it sometimes seems to maintain very well [10-13]. At the phylogenetic level, this plasticity is somewhat reflected by the population structure of *E. coli*, which is characterised by the presence of distinct phylogenetic groups (or “phylogroups”) observable by phylogenetic reconstruction [14] or the use of specific markers [15]. Four major (A, B1, B2 and D) and two minor (E and F) phylogroups have so far been described [14,16]. Judging from the non-random isolation frequencies of different phylogroups in various hosts and environments [8,9,17], it seems that the fitness in different environments varies among *E. coli* isolates from different phylogroups, which raises the question of the evolutionary nature of these phylogroups. Are they the present reflection of *E. coli* subgroups undergoing speciation as a consequence of slightly variable ecologies? Or, the primary environment of any *E. coli* being the gastrointestinal tract of endotherms, is there a relative cohesion of these phylogroups within the *E. coli* species after all? An indirect but efficient method to answer these questions is to look at the patterns of recombination (homologous and non-homologous) between different strains and members of the different phylogroups. As mentioned above, recombination should be conditioned by existing ecological differences between lineages, and may even be partly responsible for them in which case this approach also has the potential to identify the genes that play a key role in the adaptation.

**Figure 1:**
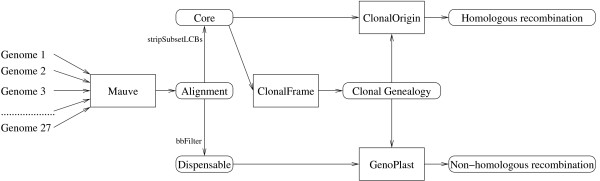
**Genomic analytical pipeline used in this study. **The genomes are first aligned using Mauve, and the core-genome is used to estimate the clonal genealogy using ClonalFrame. Non-core regions are then interpreted in terms of non-homologous recombination events on the branches of the clonal genealogy using GenoPlast, whereas core regions are analyzed using ClonalOrigin to infer homologous recombination events and their origins on the clonal genealogy.

In this study, we contribute to the understanding of the association between genomic evolution and ecological adaptation by presenting bioinformatic analyses of recombination events (gene gain/loss and homologous recombination) between 27 publicly available genomes of *E. coli* from different phylogroups (A, B1, B2 and D) and ecological backgrounds (commensal and different pathotypes). More generally, our extensive knowledge about *E. coli* compared to other microbial species provides a unique opportunity to study the mechanisms of genomic evolution in its biological context. We used a genomic analytical pipeline (summarized in Figure 1) which combined progressiveMauve [18] for aligning the genomes, ClonalFrame [19] to establish their clonal relationships with one another, GenoPlast [20] to study non-homologous recombination and ClonalOrigin [21] to examine homologous recombination.

## Methods

### Genome sequences

A total of 30 genomes of *E. coli* were available from the NCBI reference sequence database [22] when this study was initiated. Three of these genomes (UMNO26 [23], IAI39 [23] and SMS-3-5 [24]) were described as members of phylogroup D but did not cluster together in our preliminary phylogenetic analysis (Additional file 1: Figure S1). Furthermore, these three genomes showed evidence of deviation in the molecular clock rate which could have confused the analyses presented here since the models in ClonalFrame [19] and ClonalOrigin [21] assume a constant clock rate (Additional file 1: Figure S1). These three genomes were therefore excluded so that we were left with a set of 27 genomes which is summarized in Table 1. Several more genomes have recently become available on NCBI, but the complex analytical pipeline we used (Figure 1) could not easily accommodate them.

**Table 1:** Genomes used in this study

**Strain**	**Pathotype**	**Phylogroup**	**Serotype**	**Length (Kbp)**	**Citation**
ATCC8739	Commensal	A	O146	4743	[25]
HS	Commensal	A	O9:H4	4635	[26]
BL21	Commensal	A	O7	4568	[27]
REL606	Commensal	A	O7	4621	[27]
K-12/BW2952	Commensal	A	O16	4570	[28]
K-12/DH10B	Commensal	A	O16	4678	[29]
K-12/MG1655	Commensal	A	O16	4631	[30]
K-12/W3110	Commensal	A	O16	4638	[31]
IAI1	Commensal	B1	O8	4692	[23]
SE11	Commensal	B1	O158:H28	4879	[32]
55989	EAEC	B1	O128:H2	5154	[23]
12009	EHEC	B1	O103:H2	5441	[33]
E24377A	ETEC	B1	O139:H28	4971	[26]
11128	EHEC	B1	O111:H-	5363	[33]
11368	EHEC	B1	O26:H11	5689	[33]
EC4115	EHEC	E	O157:H7	5564	[34]
TW14359	EHEC	E	O157:H7	5520	[35]
EDL933	EHEC	E	O157:H7	5520	[36]
Sakai	EHEC	E	O157:H7	5490	[37]
CB9615	EPEC	E	O55:H7	5378	[38]
APEC01	ExPEC	B2	O1:K12:H7	5074	[39]
UTI89	ExPEC	B2	O18:K1:H7	5057	[40]
S88	ExPEC	B2	O45:K1	5024	[23]
CFT073	ExPEC	B2	O6:K2:H1	5223	[41]
ED1A	Commensal	B2	O81	5201	[23]
536	UPEC	B2	O6:K15:H31	4930	[42]
E2348/69	EPEC	B2	O45:K1	4957	[43]

### Multi-locus sequence typing data

**Figure 2:**
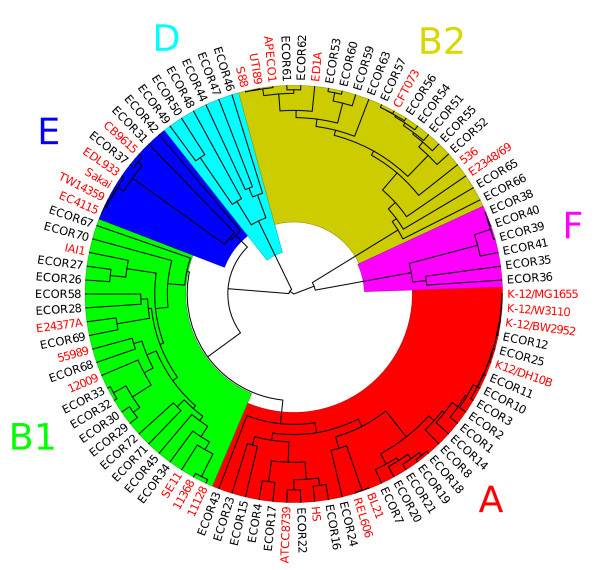
**Representativeness of the genomes used. **Phylogenetic relationships between the 27 genomes in this study (labels in red) and the ECOR reference collection (labels in black). Colors correspond to clade designations as follows: clade A in red, B1 in green, B2 in yellow, E in blue, D in cyan and F in mauve.

To assess the representativeness of the 27 strains included in this study, we compared them with the isolates from the *E. coli* reference collection (ECOR) [44] which have been characterized by two independent Multi-Locus Sequence Typing [45,46] schemes. Fragments of 450-550bp from seven housekeeping genes (*adkfumCgyrBicdmdhrecA* and *purA*) have been sequenced previously for a total concatenated length of 3423bp [47]. Additional fragments of 450-600bp from eight genes (*dinBicdApabBpolBputPtrpAtrpB* and *uidA*) have subsequently been sequenced for a total concatenated length of 4095bp [16]. To achieve maximum robustness, we combined the data from both studies to obtain 7518bp of sequence from each isolate. BLAST [48] was used to extract the sequences of each of the 15 gene fragments from each of the 27 genomes. A UPGMA dendrogram was then constructed to illustrate the phylogenetic relationship between the genomes and the ECOR collection (Figure 2).

### Analysis of genomic content

**Figure 3:**
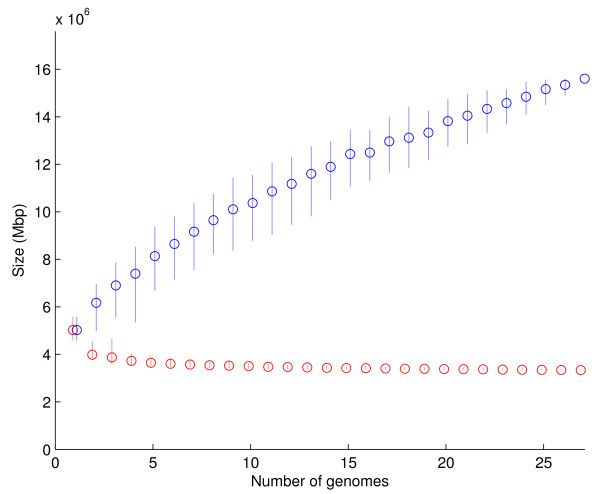
**Core and pan genome cumulative plot. **Concatenated length of the regions found in all (red) and at least one (blue) genome as more and more of the 27 genomes are aligned against altogether.

**Figure 4:**
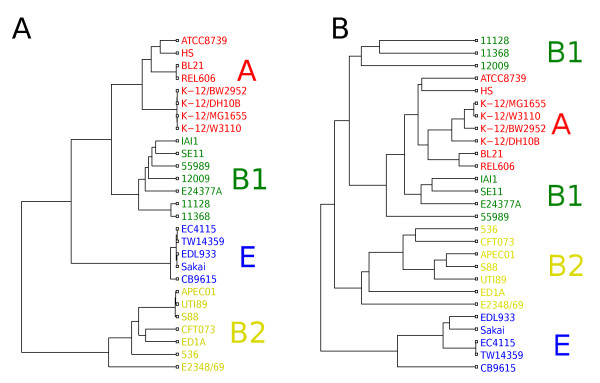
**Genealogies based on homology and gene content. **(**A**) ClonalFrame result based on core-genome. (**B**) UPGMA dendrogram based on similarity of genomic content. Colors correspond to clade designations as follows: clade A in red, B1 in green, B2 in yellow and E in blue.

The genomes of the 27 strains in Table 1 were aligned using progressiveMauve [18,49,50]. progressiveMauve does not use annotations to guide the alignment. Consequently, when there are multiple copies of a gene in the genome, progressiveMauve will usually align the copy that fits best in the context of surrounding sequence, unless the identity to a sequence in a different context scores so much better that it exceeds the breakpoint penalty. In general this will have the effect of aligning orthologous copies of genes unless the gene conversion rate among paralogs is very high. The resulting alignment contained 2675 locally colinear blocks (LCBs). For all subsets of the genomes with cardinality ranging from 1 to 27, the concatenated size of the homologous regions found in all or a fraction of the subset was counted directly from the output of progressiveMauve. These values were used to generate Figure 3. Furthermore, for each pair of strains, a pairwise distance was computed representing the proportion of genome content that they have in common. This matrix of pairwise distances was then used to build the UPGMA tree in Figure 4B. The cophenetic correlation coefficient [51] for this tree was 0.89 indicating that it is a fairly good representation of the differences in genomic content between the genomes.

**Figure 5:**
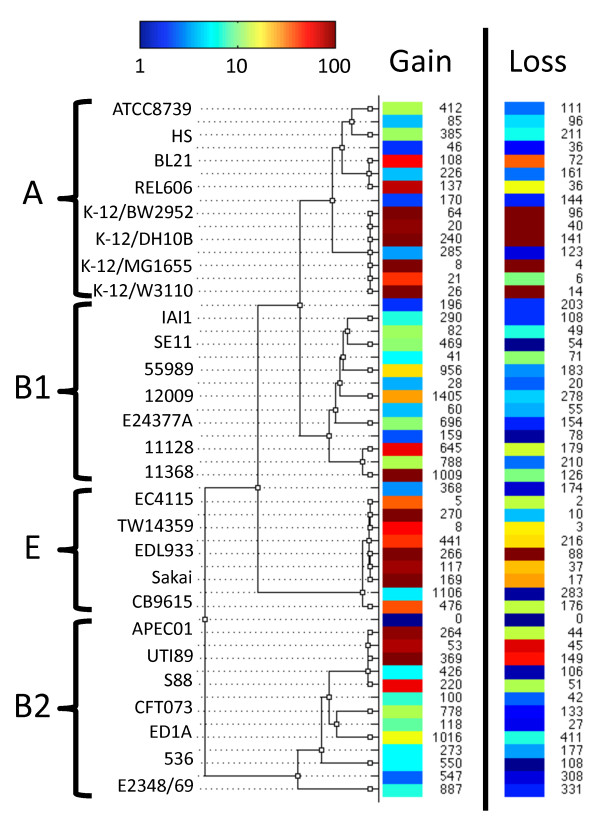
**Patterns of non-homologous recombination. **GenoPlast was used to infer how much material was gained and lost on each branch of the ClonalFrame tree. Each row corresponds to a branch of the tree as shown, gain is shown on the left and loss on the right. The numbers indicate the average amount of material gained and lost on each branch, measured in Kbp. The colors indicate how high the amounts gained or lost are relative to their expectations under a model of fixed gain and loss rates, where amounts gained and lost would be proportional to branch lengths. These colors are measured on a logarithmic relative scale as indicated at the top-left.

From the complete alignment of the 27 genomes, a matrix of feature presence/absence was computed using the bbFilter script distributed with Mauve, where each feature represented 50bp of unique sequence. This data was analyzed using GenoPlast [20] which infers how the genomic composition of the genomes evolved on the branches of the clonal genealogy (computed as explained in the next paragraph) assuming a model in which gain and loss of genetic material follow a relaxed molecular clock [52]. Briefly, GenoPlast explores the space of gain and loss events happening on branches that are compatible with the observed patterns of sharing of genomic regions observed in the genomes at the leaves of the tree. GenoPlast was run for 2,000,000 iterations with the first half discarded as burn-in. Good convergence and mixing properties were found by comparing different runs. The results of the GenoPlast analysis are shown in Figure 5.

### Reconstruction of clonal genealogy

All regions of at least 500bp found in all 27 genomes were extracted from the progressiveMauve output using the stripSubsetLCBs script distributed with Mauve. A total of 765 such regions were found, ranging in size from 501bp to 27,115bp with a mean of 4322bp and a concatenated length of 3.3Mbp. These regions found in the 27 genomes represent the core-genome of *E. coli* (Figure 3). We applied ClonalFrame [19] to this core-genome in order to reconstruct the clonal relationships between the genomes. ClonalFrame is a Bayesian phylogenetic method which performs inference under an evolutionary model accounting for the effect of homologous recombination [19,53,54]. Five runs of ClonalFrame were performed independently each consisting of 100,000 iterations, the first half of which was discarded as burn-in. The results were compared between runs and found to be highly similar, indicating good convergence and mixing properties. The clonal genealogy inferred by ClonalFrame is shown in Figure 4A. The analyses of homologous and non-homologous recombination described below were performed conditionally on this clonal genealogy. Consequently, the fact that some genomes are more closely related to one another than others is fully accounted for in these analyses.

### Analysis of homologous recombination

In order to further analyse the role played by homologous recombination during the diversification of *E. coli* from a common ancestor, we applied the computer software ClonalOrigin [21] which performs approximate inference under the coalescent with gene-conversion model [55,56]. ClonalOrigin detects recombination events, including their origin and destination on the clonal genealogy, and can therefore be used to reconstruct trends and patterns of homologous recombination [21,57]. The ClonalOrigin model rests on three global parameters which are the average length of recombination events *δ*and the scaled rates of mutation and recombination events respectively equal to *θ*_*s*_=2*N*_*e*__*μs*_ and *ρ*_*s*_=2*N*_*e*_*r* where *N*_*e*_ is the effective population size, *μ*is the per site per generation mutation frequency and *r* is the per site per generation recombination frequency. A first run of ClonalOrigin was performed for each of the 765 core regions where each region independently infers the three parameters (this phase is called “Step 2” in [21]). The median values of the three parameters across all regions were as follows: *δ*=542bp, *θ*_*s*_=0.0125 and *ρ*_*s*_=0.0128. ClonalOrigin was then rerun for each region with the three parameters set equal to these estimates (this phase is called “Step 3” in [21]). In both steps, ClonalOrigin was run for 2,000,000 iterations, the first half of which was discarded as burn-in.

**Figure 6:**
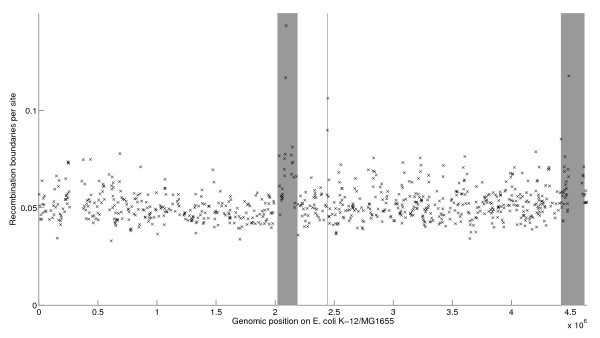
**Intensity of recombination along the genome. **Scatter plot where each cross represents a genomic region found in all 27 genomes. The X-axis indicates the positionof the region in the reference genome K-12 MG1655 [30] and the Y-axis is a measure of the intensity of recombination inferred by ClonalOrigin. Three hotspots of recombination are highlighted in grey.

**Figure 7:**
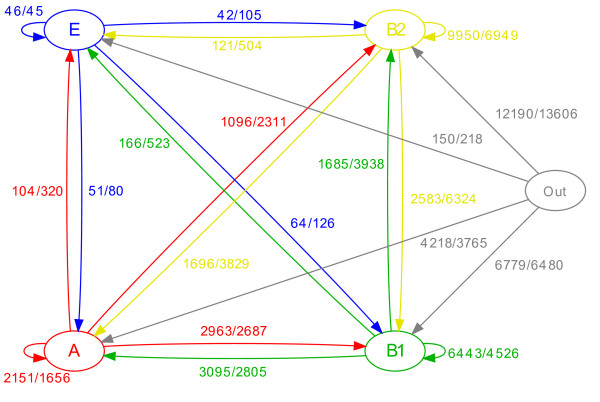
**Flux of recombination between clades. **Graph where the four clades of *Escherichia coli* A, B1, B2 and E are shown as nodes and edges represent recombination within and between them. Each edge corresponds to imports from one of the four clades (or external) into one of the four clades, and is labelled by two values separated by a forward slash. The first value is the number of recombination events inferred by ClonalOrigin. The second value is the number of recombination events expected under the ClonalOrigin model. With the exception of the flux from clade E into clade E, all observed values are outside of their expected 99% credibility intervals.

**Figure 8:**
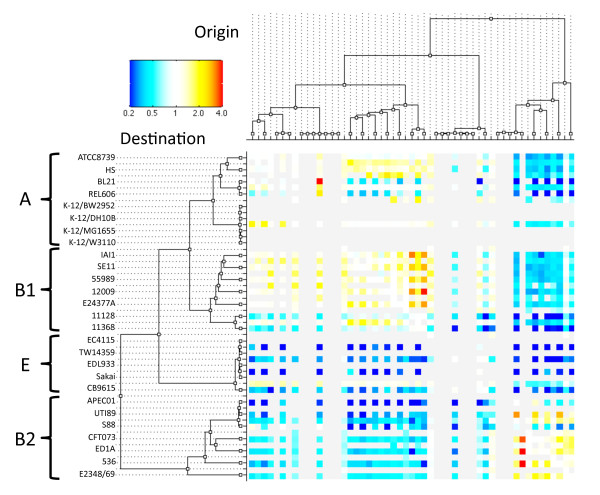
**Detailed flux of recombination. **Heat map showing the number of recombination events inferred by ClonalOrigin relative to its expectation under the prior model for each donor/recipient pair of branches. Cells for which both the number of observed and expected events are less or equal than three are shown in light gray.

Step 2 was only used to infer the values of the three global parameters, and all results presented here are based on the Step 3 results from ClonalOrigin. For instance, Figure 6 represents the number of recombination boundaries found in each of the 965 regions, with three hotspots (defined as contiguous regions of the genome in which the average recombination rate across alignment blocks is significantly higher than elsewhere in the genome) highlighted in grey. Figures 7 and 8 compare the number of inferred recombination events between different parts of the genealogy with the number expected under the prior model. These two figures are based on the numbers of the observed and expected recombination events computed by ClonalOrigin for all pairs of potential donor and recipient branches of clonal genealogy. These values are compiled in Additional file 2: Table S1.

## Results and discussion

### Representativeness of the strains used in this study

This study included 27 previously sequenced genomes of *Escherichia coli* (Table 1). To assess how representative these genomes are of the global diversity of the species, we compared them to the *Escherichia coli* reference collection (ECOR) [44] on the basis of two Multi-Locus Sequence Typing schemes which together spanned a total of 15 genes [16,47]. The resulting phylogeny (Figure 2) highlighted the six previously described lineages of *E. coli*, designated A, B1, B2, E, D and F [14,16]. Overall, the 27 strains covered much of the diversity of *E. coli*, with eight strains in clade A, seven in clade B1, five in clade E and seven in clade B2 (Figure 2; Table 1). In each of these four clades, the strains seem to represent much of the within-clade diversity rather than being closely related within the clade. However, two clades were not represented in this genomic panel: clade D and clade F. Three genomes from these phylogroups (IAI39 [23], SMS-3-5 [24] and UMN026 [23]) were initially intended to be included, but were removed because they showed evidence for significant deviation from the assumption of a fixed molecular clock (Additional file 1: Figure S1). Figure 2 indicates how the diversity of the genomes in this study relates with that of the ECOR strains, however, it should be noted that the issue of biased sampling of bacterial isolates is frequent and it is never possible to be sure of the representativeness of a sample [4,58].

### Reconstruction of the clonal genealogy

Aligning the 27 genomes using progressiveMauve [18,49,50] allowed us to compare their genomic content. As more genomes are considered in the analysis, the cumulative size of genomic regions shared by them decreased down to about 3.3Mbp, or about two thirds of each genome (Figure 3). Since this length is roughly constant whether 10, 15, 20 or all 27 genomes are aligned (Figure 3), these regions are likely to represent the core-genome of *E. coli*, which means that homologs of these regions would be found in virtually any sequenced genome. We found 765 core regions present in the genomes of all 27 strains, with total length 3,306,899bp. These core regions were input into ClonalFrame [19] in order to estimate the clonal genealogy in a way that accounts for homologous recombination which can confuse the signal of clonal inheritance [59]. This aspect is important because recombination has been reported to be frequent in *E. coli* by a large number of previous studies [14,16,47,60,61]. The inferred clonal genealogy (Figure 4A) consisted of four clades corresponding to A, B1, B2 and E. The relationships between genomes within clades were fully resolved, which is typically not achievable with MLST (eg. [14,16]). The relationships between clades were unbalanced, with clade A and B1 most closely related to each other, and clade B2 most distant from any other clade. The stemminess (ie. the ratio of internal to external branch lengths) of this tree was compatible with expectation under the standard coalescent model (Additional file 3: Figure S2), suggesting no evidence for population size variation during the evolution of *E. coli*[62-64].

### Analysis of the dispensable genome

In contrast to the core regions described above, non-core regions are found only in a strict subset of the genomes. The set of non-core regions is called the dispensable genome and together with the core genome forms the pan-genome [65-67]. The cumulative length of the non-core regions continues to increase up to the 27th genome, showing no sign of flattening, with each new genome adding about 250Kbp of previously unobserved sequence (Figure 3). This distribution has been observed before, including in *E. coli*, and its pan-genome has consequently been called “open” [23,65-67]. However, an important difference between these previous studies and ours is that in Figure 3 the lengths of genomic material are measured directly whereas previous studies counted the number of genes. Our analysis is therefore robust to the problem of identifying homologous families of genes. Nevertheless, this result indicates that the pan-genome of species with a high diversity and ecological plasticity such as *E. coli* draws from a large repertoire of genes that can be gained and lost through lateral gene transfer [5,67].

The similarity of the genomes in terms of genomic content was calculated from the patterns of presence and absence of non-core regions (Figure 4B). Compared with the clonal genealogy (Figure 4A), the clade structure is only partly preserved in this tree of genome content, with clades B2 and E intact but clades A and B1 intermingled. Clade B1 was split into three parts which were perfectly congruent with pathotypes. The three EHEC strains 12009, 11368 and 11128 [33] formed one separate cluster. The two commensal strains IAI1 [23] and SE11 [32] and the only ETEC strain E24377A [26] constituted another separate cluster, in which the two commensal strains were closest to each other. Finally, the EAEC strain 55989 [23] was on a separate branch in spite of its close relationship with the commensal strains IAI1 and SE11 in the clonal genealogy (Figure 4A). This subdivision of B1 in terms of genomic content has been partially hinted at before [68] and the fact that it is congruent with pathotypes suggests that it is linked with differences in ecological and pathogenic lifestyles. The presence or absence of genomic regions in the 27 observed genomes is the result of a process of gain and loss of content by the ancestors of the genomes since their evolution from a common ancestor. If gain and loss happened randomly and at constant rates, the tree based on genomic content (Figure 4B) would be expected to to be very similar to the tree based on homology of the core-genome (Figure 4A) since the evolution of both core and pan genomes would then follow the same molecular clock. The two trees were however highly different, indicating that the non-homologous recombination process (gain and loss of regions) did not follow a strict molecular clock. GenoPlast [20] was used to infer the non-homologous recombination events that happened in the context of the clonal genealogy inferred by ClonalFrame (Figure 4A) under a model where the rates of gain and loss are allowed to change. The results of the GenoPlast analysis are shown in Figure 5, with differences in the rates of gain and loss on specific branches spanning two orders of magnitude. The rates of gain and (to a lesser extent) loss of genomic material were found to be higher on the short recent branches within clades A, E and B2 than on older and longer branches, which explained the higher stemminess of the genomic content tree (Figure 4B) compared with the clonal genealogy (Figure 4A).

The branch directly above EHEC strain 12009 had the largest amount of gain of any branch (1405 Kbp) whereas the branch above the common ancestor of the other two B1 EHEC strains 11368 and 11128 was the highest amount of gain for an internal branch (788 Kbp; with the exception of the very long branch above clade E). Amongst the genomic material gained on these two branches, 265 Kbp were shared by the three genomes, which explained why they clustered together in Figure 4B. The distribution of this gain on the three genomes (Additional file 4: Figure S3) indicated that their convergence in genomic content happened as a result of multiple gain events that happened both on the branch above 12009 and on the branch above the common ancestor of 11368 and 11128. The convergence in genomic content of the three EHEC B1 strains was therefore reciprocal rather than unidirectional. Few convergence events were found on the branches directly above 11368 and 11128 (Additional file 4: Figure S3) in spite of considerable gain on these branches (1009Kbp and 645Kbp respectively), which could indicate that the convergence in gene content with 12009 is not on-going. Unsurprisingly, this convergence involved several genes known to be EHEC determinants, including Shiga toxins [69] and all genes from the locus of enterocyte effacement (or LEE [70]). However, it also included additional genes, such as flagellar genes (*fli*[71]) and a few metabolic clusters (*frl*[72] and *gal*[73]) with a notable presence of genes involved in aromatic compounds metabolism (*hpahpcmhp* and *mhp*[74]). These genes were not present in the other B1 strains examined in this study, which may indicate that acquiring EHEC determinants via HGT is an important means of *E. coli* adaptation, possibly enhanced by the differences in host-associated selective pressures on EHEC compared to commensals or more opportunistic pathotypes.

### Homologous recombination hotspots in *Escherichia coli*

To quantify the propensity, genomic distribution and directionality of homologous recombination during the evolution of *E. coli*, we applied ClonalOrigin [21] to the 765 core regions and assuming the clonal relationships between genomes estimated by ClonalFrame [19] in Figure 4A. The average length of fragments involved in homologous recombination was estimated at *δ*=542bp. This is almost ten times higher than a previous estimate in *E. coli*[23], but is of the same order as recent whole-genome estimates in *Bacillus cereus*[21], *Helicobacter pylori*[75] or *Chlamydia trachomatis*[76]. The relative rate of occurrence of recombination and mutation [77] was estimated at *ρ*_*s*_/*θ*_*s*_=0.0128/0.0125=1.024 which means that overall recombination happened just as frequently as mutation. The estimated rate of homologous recombination was fairly constant throughout the genome (Figure 6), with the exception of three clear hotspots (highlighted in grey) in which recombination rates were significantly higher. This included two large regions around the *rfb* operon involved in synthesis of the O antigen (positions 2,020 to 2,190 Kbp in the reference genome K-12/MG1655 [30]) and around the *fimA* gene (positions 4,420 to 4,620 Kbp). These two regions had been reported previously as hotspots of diversity and recombination [23,78].

A smaller recombination hotspot was also detected, made of just two nearly adjacent core regions (between positions 2,442 and 2,447 Kbp). This region had a similarly high recombination rate as the two regions above, but had not previously been detected as a hotspot, perhaps because of its small size (around 5 Kbp). This hotspot contained genes *yfcLyfcMyfcAmepAaroCprmB* and *smrB*. The gene *mepA* encodes for a murein endopeptidase [79] whose role is presumably to restructure the bacterial cell wall during elongation or stabilise the peptidoglycan. Mutational analyses on *mepA*[79,80] do not provide enough information to explain why recombination should be high for this gene. In the bacterial cell, *aroC* governs the synthesis of chorismate, a key precursor to the biosynthesis of aromatic compounds including the amino acids tryptophan and phenylalanine but also the siderophore enterobactin. The positive maintenance of a functional allele of *aroC* is arguably crucial for the cell to maintain appropriate levels of these amino acids and siderophores in natural conditions. In *Salmonella*[81], as well as in *Brucella suis*[82], *aroC* is required for virulence. Incidentally, *aroC* is a common target to produce knocked-out attenuated vaccine strains [83], for instance in *Salmonella* serovars Typhi [84-86] and Typhimurium [81,85,87,88], pathogenic *E. coli*[89], *Brucella suis*[82], *Burkholderia pseudomallei*[90] or *Edwardsiella tarda*[91]. To our knowledge, this is the first mention of *aroC* being part of a recombination hotspot, giving additional clues on evolutionary dynamics at this locus. Depending on how *aroC* is involved with virulence in *E. coli*, it may be under selective pressure from the immune system of the host, which could explain the observed peak in recombination rate [4], but this hypothesis will need further work to be fully assessed.

### Flux of homologous recombination

The numbers of recombination events inferred by ClonalOrigin were counted for every combination of clades receiving and donating, and these values were compared with their expectation under the ClonalOrigin model which represents a close approximation to the coalescent model with gene conversion [21,55,56]. This comparison revealed significant non-uniformity in the homologous recombination flux within and between clades (Figure 7). The three clades A, B1 and B2 had higher numbers of within-clade recombination than expected, whereas clade E had almost exactly the expected number. On the other hand, the number of recombination events detected between clades was almost systematically below its expected value, with the only exception being recombination from clade A to B1 and vice-versa which had slightly higher than expected values. Clades A and B1 are the two most closely related phylogroups (Figure 4A) which may contribute to explain this observation. Overall, inter-phylogroup recombination fluxes were lower than intra-phylogroup ones, which is compatible with the hypothesis that there is a preferred way of gene sharing within phylogroups [92]. This preferred exchange among strains of the same phylogroups could be explained by the possibility that the different *E. coli* phylogroups have slightly distinct ecological overlaps, which makes the likelihood of gene transfer higher among them than between them.

A similar analysis as above was performed on a branch-by-branch basis rather than a clade-by-clade basis (Figure 8), the only added difficulty being that some donor/recipient pairs of branches have too low numbers of expected and observed recombination events for the comparison to be meaningful (represented in grey in Figure 8).This analysis confirmed the general pattern described above, with more recombination than expected within-clades and between A and B1, and less recombination between all other clades. However, it also allowed the comparison of the individual behaviours of strains belonging to the same clade. For instance, strains BL21 and REL606 [27] showed little history of importing recombination from clade B1, contrasting with ATCC8739 [25] or HS [26] even though all four strains belong to clade A. This may be explained by the fact that these two strains are laboratory-adapted derived from *E. coli* strain B [27,93], so that they would have had little or no opportunity for recent encounter and recombination with B1 strains.The four K-12 strains in this study [28-31] were also laboratory-adapted, but had terminal branches too small to reliably estimate deviations in the number of recombination events. These four strains all originated from bioengineering manipulation on K-12 lineages over the last century and therefore harbour a very limited number of differences between them compared to what would be observed in natural populations.

The B1 strains 11128 and 11368 [33] showed significantly less sign of import from clade A (and to a lesser extent from B1) than other strains of B1. This observation implies that these EHEC strains have stopped recombining with strains of clade A (which are all commensals) as they adapted to this new pathogenic lifestyle. Two of the highest values throughout Figure 8 were the ones corresponding to imports from strains 11128 and 11386 into strain 12009 [33]. As previously noted, these are the only three EHEC strains in B1, and these three genomes have been converging in genomic content due to numerous non-homologous recombination events. This result indicates that the three genomes also have an extensive history of convergence through homologous recombination, which may have occurred at the same time as the gain of new shared genes. The evolutionary history of these three genomes seems therefore analogous to that of *Salmonella* serovars Typhi and Paratyphi A, for which both core and pan genomes converged through recombination as they were progressively adapting to exclusive infection of the human host [6].

### Speciation in *E. coli*

In the analysis of homologous recombination described above, three groups corresponding respectively to phylogroups E, B2, and A+B1 exhibited more recombination within than between one another (Figures 7 and 8). This pattern is compatible with a definition of speciation in bacteria in which recombination plays the role of a cohesive force counterbalancing divergence by genetic drift and population structure, and where species appear when this force is weakened between lineages [1,2,94]. Under such a model, patterns of genetic diversity can be generated *in silico* similar to those observed for example in *Salmonella enterica*[95,96]. The three groups might therefore represent lineages that, because of slightly distinct ecologies or notable variations in the species life cycle, have gradually diverged too far from one another for recombination to play its cohesive role, so that they might eventually become separate species, should these variations remain or increase. In other words, all *E. coli* phylogenetic backgrounds are found in the gut of endotherms [14] which is their primary environment, and to some extent in nonhost secondary environments [8,9] but it sounds plausible that phylogroup-associated variations in ecological fitness in different hosts or secondary environments could gradually decrease the physical and ecological overlap of strains from different phylogroups through time, and therefore the genetic flux between them. A number of studies seem to support this hypothesis, as different proportions of the different phylogroups are found in different environments and hosts [8,9,97,98]. Additionally, some phylogroups seem to harbour strains that have either host-restricted or more generalist lifestyles [99], as well as strains that are either resident or transients in their ability to colonize the gut [100]. Our study contributes to highlight that such variations in ecology could potentially have an impact on genetic exchange in *E. coli*.

An additional number of factors can be evoked to explain why the three groups would be diverging, including differences in their geographic distribution, adaptative selection, or simply as a result of the dependence of recombination on homology between donor and recipient [4,94,96,101,102]. The three groups are clearly separate in terms of genomic content (Figure 4B) which could explain why they recombine less with each other and why clades A and B1 still recombine frequently since they are not differentiated in terms of genomic content. To test this hypothesis, we compared the distribution of the number of recombination events found in the middle and at the edge of core regions (Additional file 5: Figure S4). We found that recombination happened more often in the middle of core regions at a small but highly significant level (Kolmogorov-Smirnov test; p-value=8.8e-09). If the variable genomic content is not just a random process, then homologous recombination would be expected to happen less often around these genes, a concept sometimes called fragmented speciation or “species in pieces” [103-106] as it would predict that speciation can apply differentially across the genome. Our results therefore demonstrate that fragmented speciation applies to *E. coli*, and that difference in genomic content is at least one of the factors driving the divergence of the three lineages.

## Conclusions

We applied a pipeline of statistical analyses in order to compare the sequences of 27 *E. coli* genomes and reveal the ancestral history of clonal relationships, homologous recombination events and non-homologous recombination events that has led the ancestor of *E. coli* to diversify into the genomes we see today. The overall picture was one of divergence between three lineages (A+B1, B2, E) which were well differentiated on the basis of both genomic content and preference for homologous recombination, with the former apparently driving the latter as expected under a fragmented speciation scenario. However, against this divergence background, we observed the convergence of three EHEC strains within B1 in both their core- and pan-genomes. These observations were correlated with the diversity of ecology and pathogenicity of the *E. coli* strains, and provide hypotheses for which genes and evolutionary processes are adaptively important.

## Competing interests

The authors declare that they have no competing interests.

## Authors’ contributions

XD, DF and AED conceived and designed the study. XD, GM and AED analyzed the data. All authors contributed to the writing of the paper and approved the final manuscript.

## Supplementary Material

Additional file 1 **Figure S1. **Test of molecular clock assumption. Neighbour-joining phylogenetic reconstruction based on all 30 genomes available from NCBI and which shows that three of them (UMNO26, IAI39 and SMS-3-5) showed significant deviation from the assumption of constant molecular clock. Click here for file

Additional file 2 **Table S1. **Detailed results of the ClonalOrigin analysis. This table contains all expected and observed values of the number of recombination events for all pairs of donor and recipient branches, as computed by ClonalOrigin. This is the data on which Figure 7 is based. There is a cell for each donor/recipient combination, and the cells are ordered vertically and horizontally in the same way as in . In each cell, two values are given separated by a semi-colon: the first one is the observed value and the second one is the expected value. Click here for file

Additional file 3 **Figure S2. **Test of ancestral population size dynamics. Distribution of expected values of stemminess under the coalescent model. The observed value for the clonal genealogy estimated by ClonalFrame is shown as a vertical line and falls within the expected values. Click here for file

Additional file 4 **Figure S3. **Gain in the three genomes 12009, 11368 and 11128. The genomic regions gained by the three genomes 12009, 11368 and 11128 are colored. The regions in red are the ones that are uniquely shared by the three genomes, whereas the regions in green are not. For genome 12009, only the gain happening on the branch directly above is shown. For genomes 11368 and 11128, the gain on the branches directly above are shown using lighter green and red, and the gain that happened on the branch above the common ancestor of 11368 and 11128 is shown using darker green and red. Click here for file

Additional file 5 **Figure S4.** Test of the fragmented speciation model. Boxplots of the distributions of the numbers of recombination events found in the middle (left) and at the edge of core regions (right). To generate the distribution on the left, the number of recombination events affecting the middle position was counted for each of the 765 core regions. To generate the distribution on the right, the number of recombination events affecting the site 10bp after the beginning of each core region was counted, as well as the number of recombination events affecting the site 10bp before the end of each core region. Click here for file
