# Supplementary figures and images for: Impact of homologous and non-homologous recombination in the genomic evolution of Escherichia coli
Source: BMC Genomics. 2012 Jun 19;13:256. doi: 10.1186/1471-2164-13-256 (PMC3505186; doi:10.1186/1471-2164-13-256)

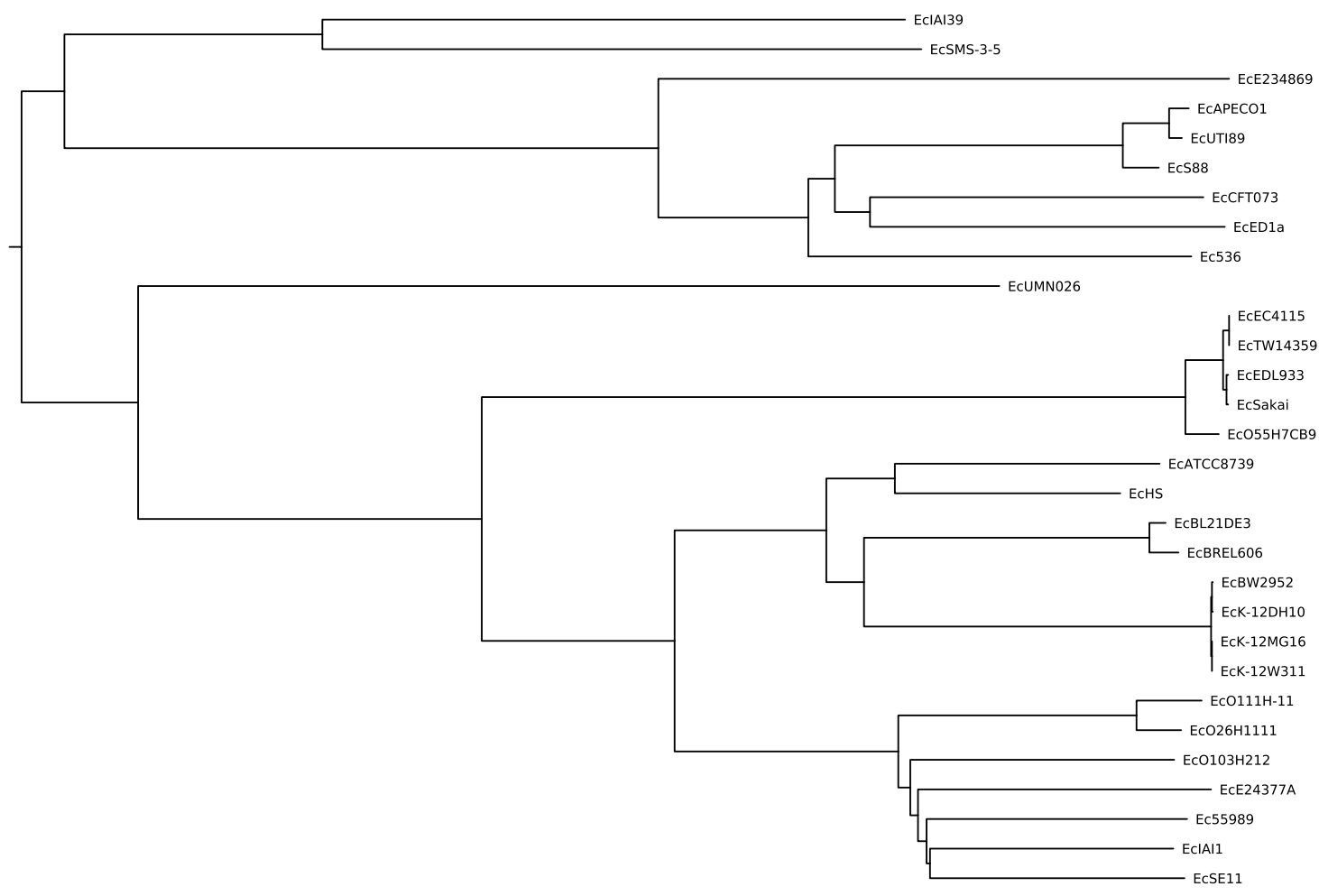

0.0030

Supplement: Additional file 1 — Figure S1. Test of molecular clock assumption. Neighbour-joining phylogenetic reconstruction based on all 30 genomes available from NCBI and which shows that three of them (UMNO26, IAI39 and SMS-3-5) showed significant deviation from the assumption of constant molecular clock. [file 1471-2164-13-256-S1.pdf]

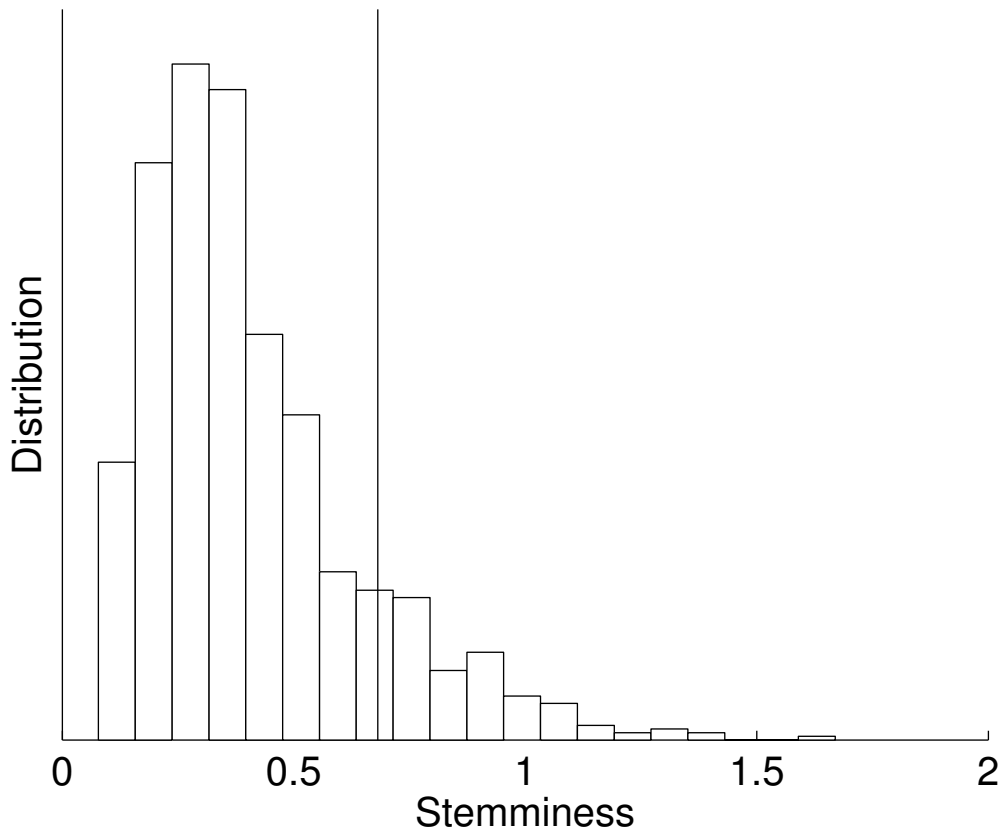

Supplement: Additional file 3 — Figure S2. Test of ancestral population size dynamics. Distribution of expected values of stemminess under the coalescent model. The observed value for the clonal genealogy estimated by ClonalFrame is shown as a vertical line and falls within the expected values. [file 1471-2164-13-256-S3.pdf]

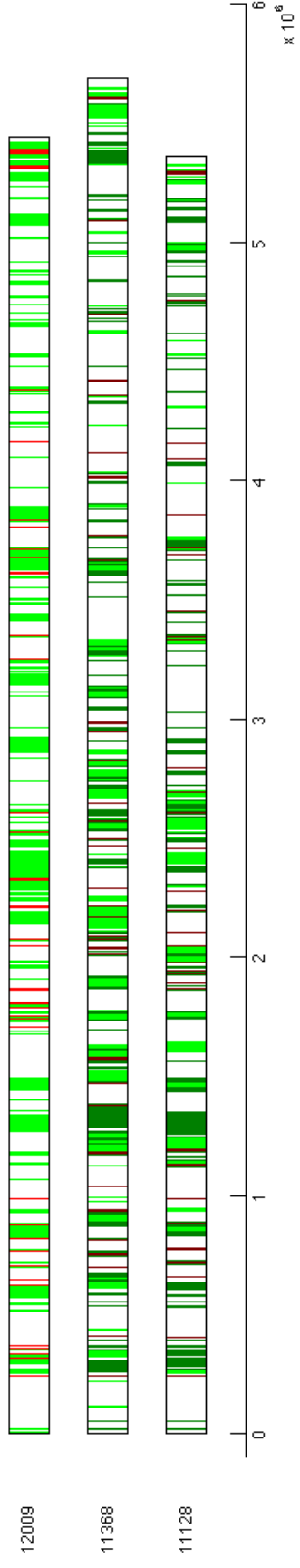

Supplement: Additional file 4 — Figure S3. Gain in the three genomes 12009, 11368 and 11128. The genomic regions gained by the three genomes 12009, 11368 and 11128 are colored. The regions in red are the ones that are uniquely shared by the three genomes, whereas the regions in green are not. For genome 12009, only the gain happening on the branch directly above is shown. For genomes 11368 and 11128, the gain on the branches directly above are shown using lighter green and red, and the gain that happened on the branch above the common ancestor of 11368 and 11128 is shown using darker green and red. [file 1471-2164-13-256-S4.pdf]

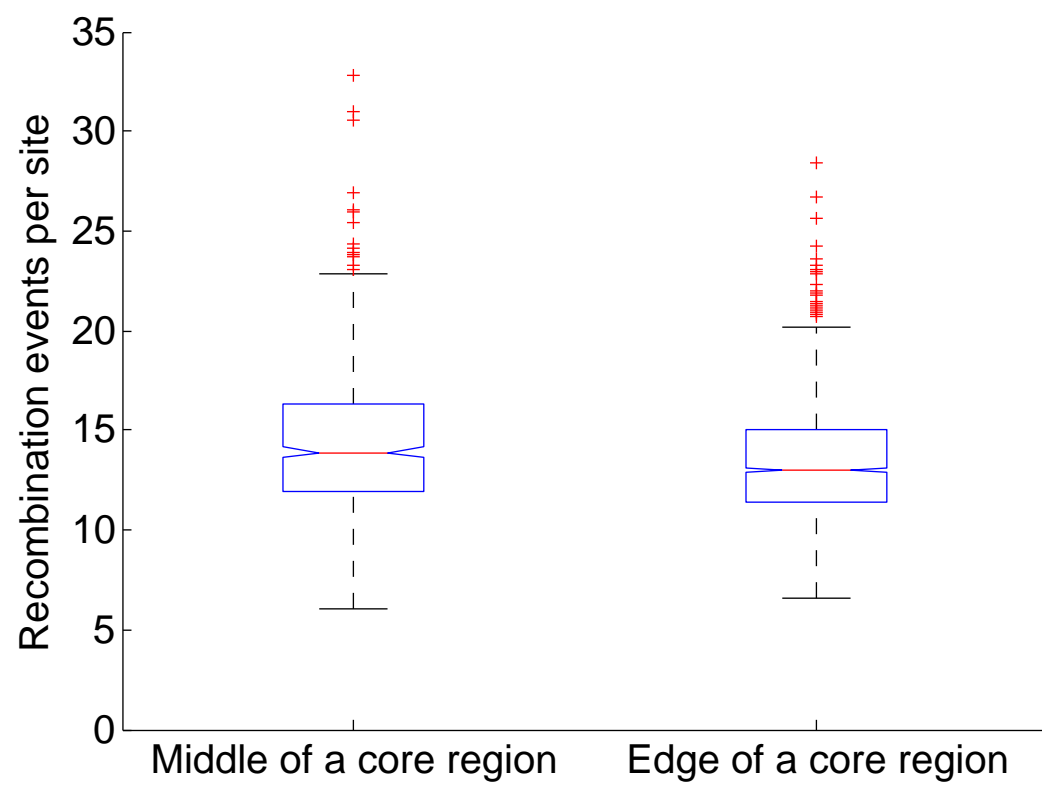

Supplement: Additional file 5 — Figure S4. Test of the fragmented speciation model. Boxplots of the distributions of the numbers of recombination events found in the middle (left) and at the edge of core regions (right). To generate the distribution on the left, the number of recombination events affecting the middle position was counted for each of the 765 core regions. To generate the distribution on the right, the number of recombination events affecting the site 10bp after the beginning of each core region was counted, as well as the number of recombination events affecting the site 10bp before the end of each core region. [file 1471-2164-13-256-S5.pdf]
